# Co-Inoculation of *Trichoderma harzianum* and *Bradyrhizobium* Species Augment the Growth of *Schizolobium parahyba* var. *parahyba* (Vell.) Blake Seedlings

**DOI:** 10.3390/microorganisms13030630

**Published:** 2025-03-11

**Authors:** Natália Cássia de Faria Ferreira, Alcides Gatto, Maria Lucrecia Gerosa Ramos

**Affiliations:** 1 Department of Forestry Engineering, Faculty of Technology, University of Brasilia, Brasilia 70910-900, DF, Brazil; alcidesgatto@unb.br; 2 Faculty of Agronomy and Veterinary Medicine, University of Brasilia, Brasília 70910-900, DF, Brazil; lucreciaunb@gmail.com

**Keywords:** consortium microorganisms, forestry production, seedling growth, synergism

## Abstract

The adoption of “consortium” of potential microorganisms can optimize the forest seedling production process. The objective of this study was to evaluate in greenhouse conditions the effect of co-inoculation between *Trichoderma harzianum, Bradyrhizobium diazoefficiens*, and *B. elkanni* on the growth of *Schizolobium parahyba* var. *parahyba* (Vell.) Blake seedlings. The treatments consisted of fungi strains (*T. harzianum* ESALQ 1306); bacteria strains (*B. elkanni* (SEMIA 5080) + *B. diazoefficiens* (SEMIA 587)); consortium (*Trichoderma +Bradyrhizobium*), and a control treatment. The seeds were sown, and evaluations were carried out 120 days after sowing. The variables analyzed were shoot height (SH), stem diameter (SD), root length (RL), shoot fresh mass (SFM), root fresh mass (RFM), total fresh biomass (TFM), shoot dry mass (SDM), root dry mass (RFM), total dry biomass (BIO), and Dickson quality index (DQI). The evaluated microorganisms proved to be effective in the production of *S. parahyba* var. *parahyba*, with emphasis on co-inoculation for growth parameters, promoting an increase in SH (23%), SD (36%), and RL (84%). For mass, non-inoculated seedlings (control) obtained a decrease of 67% (TFM) and 83% (BIO) compared to co-inoculation. The results indicate a promising method in seedling production; the biostimulators allowed the increase in plant development, which led to success in the morphometric indices. The mechanisms involved in the co-inoculation of microorganisms’ consortium in promoting the growth of native wood species to allow their production on a large scale in the silvicultural sector are still scarce, and new research is needed to elucidate the physiological and biochemical mechanisms involved.

## 1. Introduction

Forestry is a consolidated segment in several global regions, mainly in the timber sector, and is supplied by cultivating seedlings grown in nurseries. In Brazil, native species meet the growing demand for large-scale production. However, a large amount of Brazilian soils have a high degree of weathering and acidity (high concentrations of aluminum (Al) due to reduced pH), and low content of available mineral nutrients, which are limiting factors to producing woody species [1,2]. Due to the restrictions of tropical soil, the synergism between plants and beneficial microorganisms contributes to improving soil structure to increase plant development [3,4].

The Brazilian planted tree sector boosts the national economy; however, the success of establishing forest seedlings depends on multiple factors, such as soil quality and the capacity for development and establishment [5]. Generally, one of the main obstacles to the establishment of forest seedlings in nursery and field conditions is correlated with the absence of potential symbiotic microorganisms in the soil, which highlights the great relevance of symbiosis in seedling production [6].

Among the potential species for forestry production, *Schizolobium parahyba* var. *parahyba* (Vell) Blake (guapuruvu) is a pioneering and native tree of the Atlantic Forest and is a promising tree, being 40 m tall and showing 120 cm DBH, for the silvicultural sector [7]. This legume has potential in ecological restoration and wood production, such as the production sectors of ships, sheet metal, furniture, doors, ceilings, tables, and toys [8]. The species requires healthy soils, with conditions conducive to the adequate development of seedlings, through improvements in soil quality and greater nutritional availability, using potential microorganisms [9,10,11].

Bio-inoculation with symbiotic microorganisms, for example, from *Trichoderma* sp. and *Bradyrhizobium* sp., can enable the production of seedlings under such limiting conditions [12,13]. The stimulating action on plant growth is complex, carried out via interactions with biochemical factors and the production of various enzymes and compounds beneficial to plants [14]. The high quality of the seedlings guides the achievement of success in the establishment of commercial forests; however, the conditions of nutritional restriction in the soil substrates commonly exist in the production of seedlings, decreasing the quality of them.

*Trichoderma* promotes the development of plants, with plant protection mechanisms against biotic (antagonism, parasitism, and/or competition) and abiotic (resilience of water deficit and temperature) stresses, and biostimulation mediated by the signaling of chemical compounds, responsible for increasing plant growth parameters [15,16,17,18]. In the production of woody species, there is a restriction of research that can validate the use of the symbiotic fungus during the growth processes. *Bradyrhizobium* is widely known for its applications in annual legume species, primarily correlated with biological nitrogen fixation (BNF) [19]. However, the capacity of stimulating bacteria overcomes development-inducing stimuli, which enhances the choice of potential bacteria in the production of leguminous tree species, not for nodulation but for growth-promoting compounds, which is an innovative, sustainable perspective [20].

Plant co-inoculation can have three aspects, beneficial, antagonistic, or null, through the high variability during the microbial association mechanism [21]. According to the broad effects on biostimulation of plant growth, recently, the growing global supply of bio-inputs has become increasingly stronger with applications to different species of beneficial microorganisms responsible for consistent, fast, satisfactory results, which are alternatives to the excessive use of fertilizers [22,23].

From a sustainable perspective, the consortium between endophytic microorganisms tends to meet the demand for higher silvicultural production rates, combined with the premise of reducing environmental impacts caused by the unrestrained use of fertilizers. The objective of this study was to evaluate the co-inoculation between fungi and bacteria through the fungal strain of *Trichoderma harzianum* and the complex of bacterial strains (*Bradyrhizobium diazoefficiens* + *B. elkanii*) in promoting growth during the production of guapuruvu seedlings (*Schizolobium parahyba* var. *parahyba* (Vell.) S. F. Blake).

## 2. Material and Methods

### 2.1. Description of the Experimental Site

The experiment was conducted in a tropical region located in Ipameri, Goias, Brazil (17°42′59.60″ S, 48°08′39.52″ W, 797 m), under greenhouse conditions between August/2022 and November/2022.

### 2.2. Soil Characteristics: Correction and Fertilization

The soil used in the experiment was a Dystrophic Oxisol-Red-Yellow according to Soil Taxonomy. Soil samples were collected from the surface layer (0–20 cm) in a native savannah in the municipality of Ipameri, Goias, Brazil. A composite sample composed of six sampling points was used for physical soil chemicals. Table 1 describes the chemical attributes before installation. Based on the fertility limitations existing in the soil, the correction was limestone (6.26 g pot^−1^), gypsum (8.10 g pot^−1^), and chemical fertilization (2.77 g pot^−1^), with the formula 04-28-08 (NPK).

### 2.3. Seeds (Schizolobium parahyba var. parahyba): Overcoming Dormancy

The germination of seeds was above 70%, providing suitable conditions for their development. A dormancy is classified as an integumentary type, with a thick external layer that aims to protect the seed from stressful conditions (biotic or abiotic). There was mechanical scarification out on the side opposite the hilum, with the aid of nº 80 sandpaper. After abrading, the seeds were placed in a sanitized and sterilized container of two liters of distilled cold water for 24 h to penetrate water into the seeds and enable the imbibition process.

### 2.4. Biostimulating Microorganisms: Soil Treatment

The commercial strains used in the present study were *Trichoderma harzianum* ESALQ 1306; SEMIA 5080: *Bradyrhizobium diazoefficiens* + SEMIA 587: *Bradyrhizobium elkanii*. Approximately 30 days after soil correction and fertilization, in plastic pots with a capacity of 10.0 L, and a dose of 8 mL of bacterial formulation (*B. diazoefficiens* + *B. elkanii*), *T. harzianum* suspension was distributed in the topsoil of each pot, totaling 4.0 × 10^8^ CFU (colony forming unit) per pot. The proposed suspension (4.0 × 10^8^ CFU) was calculated for each commercial strain evaluated to determine the equivalence of product concentration, both for the *Trichoderma* strain and for *Bradyrhizobium*, according to the dose adjustment, for equal amounts of CFU, considering that each bio-input used (strains) has specific concentrations.

The suspensions were distributed superficially in pots, in the morning, and the *S. parahyba* var. *parahyba* seeds were manually sown (5 seeds per pot, and thinning was performed to one seed per pot when the plants reached 4–5 cm in height). The experiment was arranged in a completely randomized design (CRD) with six replications (pots) for each treatment (commercial strain). For comparative purposes, treatment without inoculation with microorganisms was included as a control.

### 2.5. Conducting Experimental Analysis

After 120 days of sowing (DAS), estimating with the aid of a graduated tape measure and a digital caliper, the following parameters were evaluated: shoot height (SH), stem diameter (SD), root length (RL), shoot fresh mass (SFM), root fresh mass (RFM), total fresh biomass (TFM), shoot dry mass (SDM), root dry mass (RFM), and total dry biomass (BIO). Subsequently, on a digital scale, total fresh mass (TFM) and total dry mass (BIO) were quantified, and the Dickson quality index (DQI) was calculated, which was determined according to the formula [24].

$$DQI=\frac{BIO}{SH\;(cm)/SD\;(mm)/SDM\;(g)/RFM\;(g)}$$

### 2.6. Statistical Analysis

The data were subjected to analysis of variance (ANOVA) and the means were compared using the Scott–Knott test (*p* ≤ 0.05). After verifying the assumptions of normality and homogeneity of residual variances of the data, analysis of canonical variables (multivariate) was carried out to evaluate the similarity of treatments by means of graphic dispersion, and multivariate analysis of variance was performed (MANOVA). Statistical analysis was performed using the “candisc” package of software R (v 4.3.3).

## 3. Results

The seedlings with the higher SH were obtained in the treatment with co-inoculated strains (*T. harzianum* + *B. diazoefficiens* + *B. elkanii*) and the strain of *T. harzianum* 1306, while the treatment with bacterial strains (*B. diazoefficiens* + *B. elkanii*) did not result in increases in shoot height, equaling the control treatment, with an approximate difference of 20% (Table 2).

Regarding the RL of the seedlings, the co-inoculated strains and the isolated strain of *T. harzianum* stood out, corresponding to an increase of around 84% and 74%, respectively, compared to the control treatment (Table 2). The SD presented similar results with the shoot height of the seedlings, with co-inoculation and inoculation with *T. harzianum*; in contrast, the treatments referred to with less influence were compared to the control, with an approximate decrease of 37%, and the *B. diazoefficiens* + *B. elkanii* strain, with an approximate reduction of 17%. These results showed promising findings (Table 2).

For SFM, co-inoculation maintained a better standard of efficiency in seedling production, reaching an average relative increase of 67% in relation to the control treatment. The other treatments demonstrated in this paper showed lower SFM, not differing from each other, and the control treatment had the lowest efficiency for accumulating TFM (Table 2). The RFM, the consortium, and the *T. harzianum* 1306 strain showed promise; in contrast, there was a reduction in accumulation for the bacterial strains (−45%) and control treatment (−77%).

The increase in SDM and RDM was significantly influenced by the interaction system between fungus and bacteria, compared to the control treatment, with a proven inferiority in biomass accumulation of around 83% for both variables. The BIO was significantly influenced by the interaction system between fungus and bacteria, with an increase of 20% and 49% in relation to the *T. harzianum* strain and *B. diazoefficiens* + *B. elkanii*, respectively. At the same time, seedlings without inoculation (control) were not able achieve the desired efficiency, which resulted in a decrease of 82%, compared to the co-inoculation treatment (Table 2). The treatment with co-inoculation showed the highest DQI, with an increase of 25% to 40% compared to *B. diazoefficiens* + *B. elkanii* and *T. harzianum*, respectively. The control treatment was represented by the lower potential of seedlings, showing a decrease of 84% in microbial consortium (Table 2).

**Figure 1:**
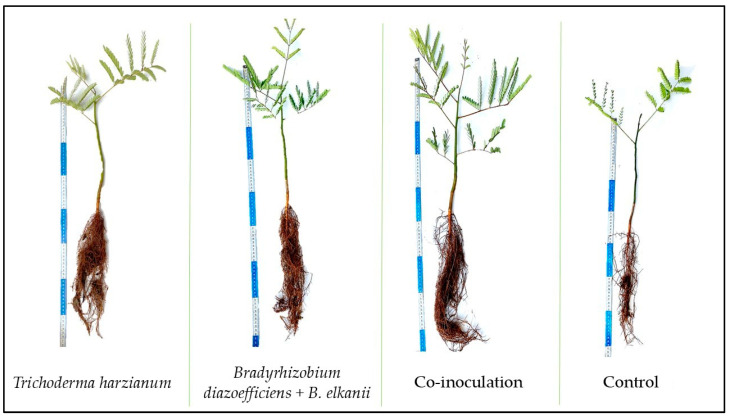
*Schizolobium parahyba* var. *parahyba* seedlings, under the effect of different inoculation of microorganisms and control treatment.

Phenotypically, *S. parahyba* var. *parahyba* seedlings stood out in relation to the increase in the root system and plant height (Figure 1). The inoculation with *T. harzianum* and co-inoculation correlated with the balance between seedling growth variables.

**Figure 2:**
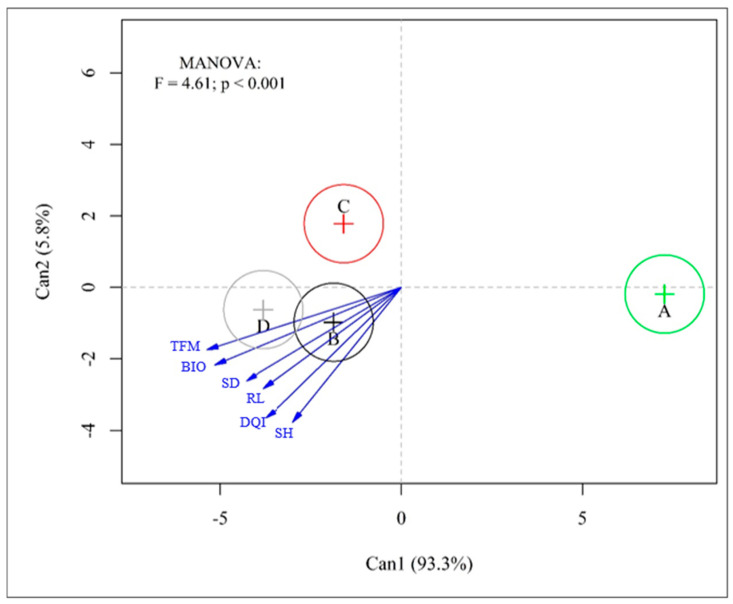
Multivariate statistical analysis (analysis of canonical variables) showed a relationship between growth parameters and seedlings of *Schizolobium parahyba* var. *parahyba* treated with suspension of microorganisms and co-inoculation. A: control; B: co-inoculation; C: *Bradyrhizobium diazoefficiens* (SEMIA 5080) + *B. elkanii* (SEMIA 587); D: *Trichoderma harzianum* ESALQ 1306. Shoot height (SH), stem diameter (SD), root length (RL), total fresh biomass (TFM), dry biomass (BIO), and Dickson quality index (DQI).

The canonical seedling variables were positively related to the analyzed variables, elucidating 99.10% of the variation in the analyzed data (93.30% and 5.80%) (Figure 2). The highest relationship obtained for the analyzed variables (SH, SD, RL, TFM, BIO, and DQI) during seedling production was obtained both by the isolated inoculation of *T. harzianum* and by the co-inoculation between *T. harzianum* + *B. diazoefficiens* + *B. elkanii* (Figure 2). However, the lowest relationship was obtained in the absence of inoculation (control treatment), followed by the *B. diazoefficiens* + *B. elkanii* strain (Figure 2).

## 4. Discussion

The results support our research hypothesis regarding the possibility of increasing the growth promotion of *S. parahyba* var. *parahyba* seedlings in the nursery phase via inoculation with beneficial microorganisms. The success of communication and interaction between plants and microorganisms is intrinsic. The evaluation of the potential of the consortium process between fungi and bacteria constitutes a promising tool for the development of forest seedlings. Despite multifunctionality, the verification of symbiotic results is not usually easy, considering the high level of specificity involved between strains. The compatibility of strains can probably explain the results obtained. There are few studies evaluating the compatibility between *Trichoderma* and *Bradyrhizobium* in woody species.

The results obtained can possibly be explained by the compatibility of strains. There is a shortage of studies evaluating the compatibility between *Trichoderma* and *Bradyrhizobium* in woody species. A morphological attribute of great importance in the evaluation of seedling production is the diameter of the stem, as it confronts the balance between the root and biomass, which will respond with plant growth. A well-developed root system can absorb and transport water and nutrients to other plant organs, correlated with the photosynthetic capacity and production of vital energy for the plant’s development.

The mechanism that possibly clarifies the observed interaction evidenced in increases in plant shoot height and root system involves root exudates into the rhizosphere, stimulating the development of biopromotion. From the connection, communication was established with the plant root system, which was responsible for compatibility between strains [25,26]. The secretion of primary metabolites (organic acids, amino acids, carbohydrates, vitamins) and secondary metabolites (alkaloids, phenolics, flavonoids, terpenoids) is attributed to the exchange of signaling molecules, due to successful association, in plants which provide energy sources such as carbohydrates/sugars [27,28].

The treatment by co-inoculation is evidenced by the compatibility between fungal and bacterial strains, with effects evidenced in the production of *S. parahyba* var. *parahyba* seedlings and an increase of 35% with the additional strains. The control treatment was represented by the low potential of seedlings, with a decrease of 84% in the microbial consortium.

Based on the morphological variables, the DQI indicates the robustness and potential of the seedlings. The guapuruvu seedlings, according to the quality indicator (DQI), point to the effectiveness of the multi-dependent effects linked to the vast diversity and complexity of the intercropped interactions. What explains the variable performance is attributed to the different metabolic pathways, which trigger specific mechanisms of action to promote plant growth, such as higher induction of photosynthetic activities; they are responsible for producing photoassimilates and the consequent success of plant growth and greater availability of essential nutrients for plant physiological functions, among other factors [29,30].

Although there are several studies regarding co-inoculation with annual legumes, symbiosis with leguminous tree species is scarce, considering that hormonal communications and chemical messengers released by the root determine the dynamics of the interaction. The results observed in Figure 1 are probably related to the effects on the cycling of organic material via decomposition, mineralization, or microbial immobilization, or through chemical reactions in the soil–plant system, such as changes in rhizospheric pH and sorption/desorption, in addition to the ability to synthesize hormones.

Rhizobacteria are highly evolved organisms, capable of producing growth-regulating enzymes in addition to stimulating the production of phytohormones, such as the growth of secondary roots and root hairs, leading to a consequent increase in the absorption of water and nutrients [31]. Hormones are “chemical messenger” regulators of physiological processes; examples are auxins, responsible for cell division and differentiation, cytokinins, which play a role in cell proliferation, and gibberellins, known as growth hormones, directly acting on the stem diameter, root elongation, and biomass accumulation [32,33].

Presumably, among the multiple benefits associated with microbial consortia, one of the indirect mechanisms possibly correlated with the effects on promoting the growth of tree legumes is biotic stress conditions, which are correlated with the protection apparatus. The effects attributed to synergistic microorganisms include the dynamism surrounding antibiosis, competition, or parasitism of phytopathogenic agents, qualified for delineating a rhizospheric environment favorable to root growth and stress-free conditions [34,35].

Notoriously, the consortium is an innovation in the forestry sector and is evidenced by the positive action between rhizobacteria in promoting plant development, as obtained for the growth of *Swietenia macrophylla*, via physiological strengthening of the root system [36]. In *Tectona grandis* seedlings, co-inoculated with (*Rhizophagus clarus* + *Bacillus subtilis*), there were improvements in nutritional absorption and improvement of up to 11.04% in the growth rate in the field [37]. Scientific evaluation obtained in eucalyptus (*Corymbia citriodora*) strengthens the effect of co-inoculation between *Trichoderma* sp. and *B. subtilis* [38].

The combined inoculation (arbuscular mycorrhizal fungi (AMF) + *Azospirillum* + *Azotobacter*) obtained satisfactory biometric results in the legume *Santalum album*, whose increase in plant height was 52.50 cm (180 days), a direct phenotypic indicator of development; the root diameter parameter was promising under the effects of microorganisms (4.27 mm at 180 days) and, together, the root length reached up to 26.00 cm (180 days) [39].

Among the factors limiting silvicultural development is the initial stages of seedling formation growth. Seedlings of inferior quality can have a low increase in biomass and restrictions in the root system, with a direct association with the low nutritional availability of the soil (substrate) used for seedling growth. The co-inoculations between *Rhizoglomus fasciculatum* (arbuscular mycorrhizal fungus), *Mortierella* sp. (mineral solubilizing fungus), and *Azospirillum brasilense* (plant growth-promoting bacteria) promoted better potential in the development of seedlings with higher quality [40].

During the nursery phase, broad factors were found to have a positive influence on the production capacity of plants, such as the effects of promotion of growth in height and the root system, and the use of this technology (bio-inputs) is viable for obtaining seedlings of native plants with a high level of quality through the “consortium” between microorganisms. Such information was reported in evaluations through the successful production of *T. grandis* seedlings under co-inoculation between mycorrhizal fungi and rhizobacteria [41].

The development stages (germination, seedling development, and seedling growth) constitute the most vulnerable phases of tree species, as they encompass one of the periods of the greatest nutritional demand. In this sense, the introduction of combined bio-inputs (fungus and bacteria) promotes beneficial effects on the vigor of seedlings, root growth, greater photosynthetic capacity (due to the formation of energy from the supply of nutrients), and obtaining high levels of biomass, which correlates with the maintenance and ecological balance of the soil, as the effects were triple: (1) vigorous seedlings; (2) soil fertility; (3) low-cost and multifunctional microorganisms.

**Figure 3:**
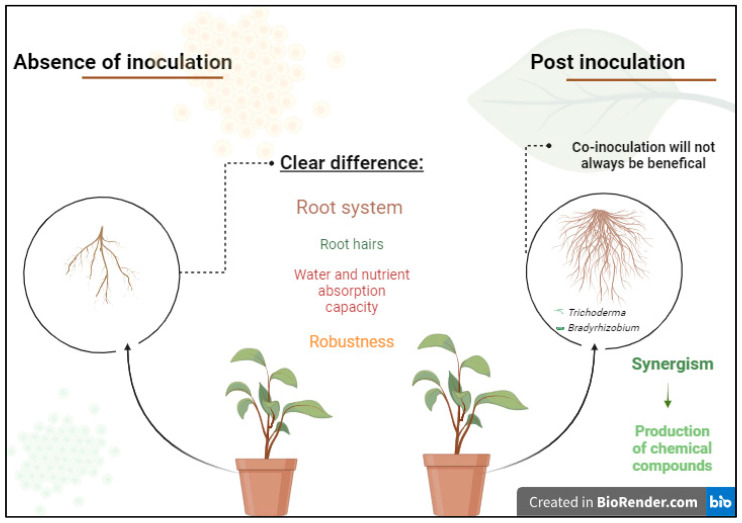
Effects on the growth plants of co-inoculated (*Bradyrhizobium* and *Trichoderma*) and non-inoculated plants.

A new possibility to reduce the cost and dependence on high fertilizer content is assigned by the action of potential microorganisms. In response to the questions involved in the study, it is possible to verify that there is an increase in production and lower production costs in the silvicultural sector associated with co-inoculation with potential microorganisms. The benefits observed in the short, medium, and long term were key factors in the production segment of wood species (Figure 3). The symbiotic association between plants and microorganisms will promote benefits in the formation of seedlings; nevertheless, this does not become a rule to find success in terms of plant–microorganism association.

The bacteria’s *B. diazoefficiens* and *B. elkanii* showed different effects when inoculated alone and in consortium. The exchange and perception of chemical signals (extensive and complex dialogue) allow or restrict the induction of compounds responsible for successful growth promotion, such as volatile organic compounds (VOCs), secondary metabolites (SMs), and plant hormones (IAA) [42,43,44], which reinforces the need to evaluate the interaction of microorganisms and species, to guide and improve the use of viable silvicultural techniques to boost the production of vigorous seedlings.

**Figure 4:**
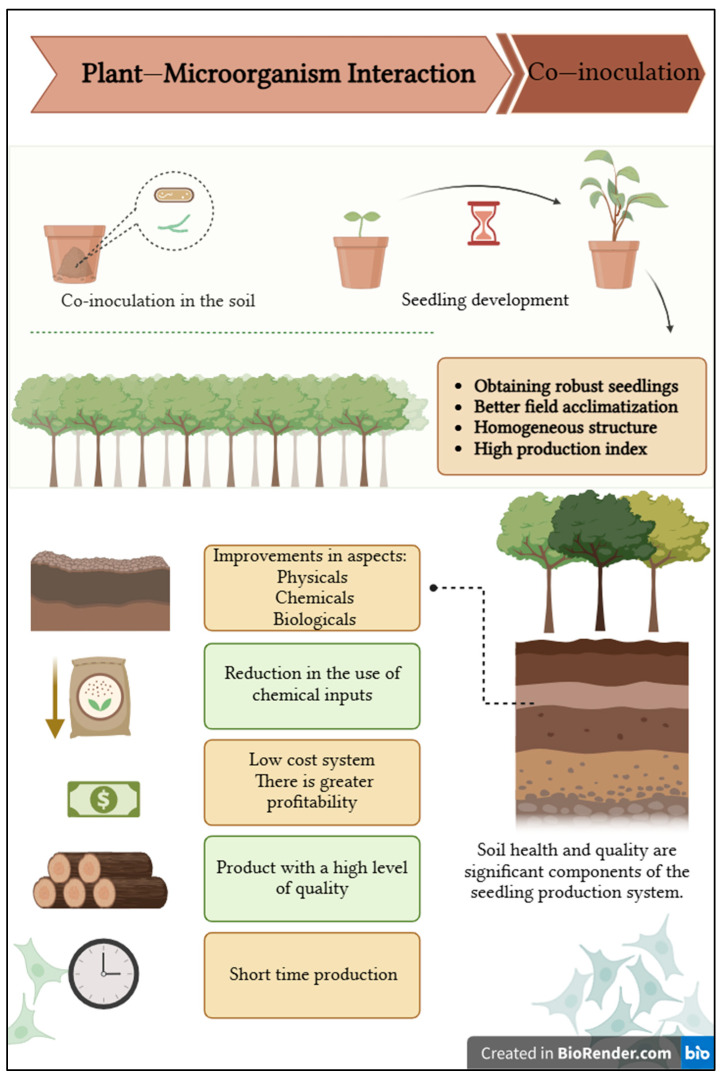
Benefits related to the adoption of bio-inputs in the production of forest seedlings.

The growing and alarming decline in soil quality and vitality reinforces the search for products from planted forests and the mitigation of exploratory processes in natural ecosystems. Soil biological fertility is a key factor in increasing the resilience and robustness of seedlings, even those resistant to external stresses. The formation of planted forests begins with the production of vigorous seedlings, supplemented by biostimulants co-inoculated in the soil, with direct effects on plant development, improvements in the physical–chemical–biological attributes of the soil, reduction in the use of chemical inputs, to guarantee the achievement of a low-cost silviculture system (production or reforestation) with shorter production times, robustness, a high level of quality, good acclimatization in the field, and the formation of homogeneous, highly productive forests (Figure 4).

In the literature, studies reinforce the results obtained in the present study, where the microorganisms involved in the production system are competent in meeting the requirements during the plant formation phase, aimed at producing high energy content via the production of photoassimilates (photosynthetic capacity), intended for plant growth [45,46]. The results obtained reveal the success of consortium between microorganisms. However, there will not always be positive effects on tree production parameters, as promotion via double inoculation can be variable, depending on the level of compatibility between microorganisms and the amount of inoculum applied (population of microorganisms), leading to antagonistic effects that are undesirable for production and sustainable development purposes.

The root system has high plasticity [47]. From there, questions arise, such as what would be the most promising way to change the roots? From a sustainable perspective, the answer correlates with biological research, increasing the chances of successful establishment in the field. Better elucidation at the species level, such as the native tree legume (*S. parahyba* var. *parahyba*) and combined strains (fungus and bacteria), proved the symbiotic success of “phytostimulation” under greenhouse conditions.

Co-inoculation has a triple effect, which transposes the growth parameters of *S. parahyba* var. *parahyba* seedlings, permeates the economy in terms of the reduction in phosphate fertilizers, and solidifies sustainability in terms of maintenance and conservation of soil quality, with the results obtained by the research being a strong starting point, evidenced by the possibility of interaction between forestry and microorganisms.

The large deficit of scientific research on native forest species compromises considerable advances in production systems, with co-inoculation being an area of innovation for large-scale and short-term production, with an impact on the forestry market and reforestation projects; it is suitable for adverse environments, competitive in terms of space (weeds and others), and successful in the search for vital resources (light, water, and nutrients) and adapting to climate fluctuations.

Concise scientific studies that evaluate the careful behavior of microorganisms to reduce dependence on plant chemical fertilization are limited. The data obtained suggest improvement in terms of the chemical mechanisms of biostimulators, associated with the singularities of the cultivation condition, affected by the conditions of water availability (irrigation or rain), luminosity indices (length of day, location, and intensity), and other factors that may affect the performance of growth-stimulating microorganisms.

## 5. Considerations and Future Perspectives

The growth of *Schizolobium parahyba* var. *parahyba* seedlings proved to be closely synchronous with the interaction between the fungus *Trichoderma harzianum* and the bacteria *Bradyrhizobium diazoefficiens* and *B. elkanii*, which stood out for their positive impact in growth parameters.

In addition to the low production cost involved, there is simplicity involved in handling and post-application effectiveness, through stimulation of root development, greater nutritional absorption capacity (previously in a format unavailable to plants), and the increase in biomass; this creates a, decisive precedent for obtaining seedlings in a highly vigorous way. DQI indicates the good development of seedlings.

Endophytic fungi associated with rhizospheric diazotrophic bacteria are beneficial, especially in the strategic context, where there can be viable production of seedlings of forest species, with profitable segments (economic gains) for commercial greenhouses designed both for the forestry market and for projects aimed at recovering degraded environments.

Co-inoculation promotes satisfactory results in the production of guapuruvu seedlings and fits within the sustainable perspective (one of the pillars of production systems). In this context, it represents a considerable reduction in the use of industrial fertilizers, as well as minimizing the exploitation of limited sources of nutrients. A “simple step” can open doors to using beneficial microorganisms in forestry production and open space for future research to evaluate the development and establishment of plants in a field environment (transplanting).

The number of studies correlated with co-inoculation compatibility is increasing. However, the number of species of trees being studied is still limited. Due to the great relevance and capacity to promote growth, there are still questions to be elucidated regarding the physiological mechanisms involved in the growing of potential native wood species to enable their large-scale production, since information correlated with the action of microorganisms in the silvicultural sector is still scarce, which requires greater attention.

## Figures and Tables

**Table 1:** Chemical characteristics of the red-yellow latosol.

**S**	**P Mehlich**	**K**	**Na**	**Zn**	**Cu**	**Fe**	**Mn**	**Ca**	**Mg**	**Al**	**H+Al**
**mg dm^−3^**				**cmol_c_dm^−3^**							
2.00	0.50	15.00	3.80	0.50	0.60	23.40	8.10	0.20	0.10	0.00	2.40
**SOM**	**Organic Carbon**	**pH**	**Sand**	**Clay**	**Silt**	**CEC**	**V**	**Ca/CEC**	**Mg/CEC**	**K/CEC**	**H+Al/CEC**
**g kg^−1^**		**CaCl_2_**	**Texture (g kg^−1^)**			**cmol_c_dm^−3^**	**%**				
8.00	4.64	5.10	370.00	540.00	90.00	2.76	12.91	2.00	3.62	1.45	86.96

Extractors: P and K Mehlich^−1^; Ca-KCl 1 mol/L; H+Al–calcium acetate 0.5 mol/L at pH 7. SOM: soil organic matter.

**Table 2:** Shoot height (SH), root length (RL), stem diameter (SD), shoot height fresh mass (SFM), root fresh mass (RFM), total fresh mass (TFM), shoot height dry mass (SDM), root dry mass (RDM), total dry mass (BIO), and the Dickson quality index (DQI) of *S. parahyba* var. *parahyba* in a greenhouse, with soil from pots treated with the suspension of *T. harzianum*; *B. diazoefficiens* + *B. elkanii* and co-inoculation (*T. harzianum* + *B. diazoefficiens* + *B. elkanii*).

Treatment	SH	RL	SD	SFM	RFM	TFM	SDM	RDM	BIO	DQI
	cm		mm	g						
*T. harzianum*	20.18 a	43.10 a	5.34 a	6.18 b	4.53 a	10.71 b	2.98 b	2.00 b	4.99 b	0.93 b
*B. diazoefficiens + B. elkanii*	16.10 b	31.80 b	4.56 b	5.80 b	3.20 b	9.00 b	2.04 c	1.54 b	3.58 c	0.74 b
Co-inoculation	19.10 a	45.40 a	5.28 a	7.18 a	5.83 a	13.04 a	3.51 a	2.71 a	6.22 a	1.24 a
Control	15.50 b	24.60 c	3.88 b	3.01 c	1.35 c	4.36 c	0.61 d	0.45 c	1.06 d	0.20 c
CV%	14.06	25.91	10.11	9.71	33.60	13.47	16.31	34.61	19.27	24.25
SE	0.64	1.69	0.17	0.37	0.27	0.48	0.25	0.80	0.49	0.15

Means followed by the same lowercase letter in the same column do not differ significantly by the Scott–Knott test (*p* ≤ 0.05). SE: standard error.

## Data Availability

The data presented in this study are available upon request from the corresponding author.
